# A flexible neural implant with ultrathin substrate for low-invasive brain–computer interface applications

**DOI:** 10.1038/s41378-022-00464-1

**Published:** 2022-12-25

**Authors:** Zhejun Guo, Fang Wang, Longchun Wang, Kejun Tu, Chunpeng Jiang, Ye Xi, Wen Hong, Qingda Xu, Xiaolin Wang, Bin Yang, Bomin Sun, Zude Lin, Jingquan Liu

**Affiliations:** 1 National Key Laboratory of Science and Technology on Micro/Nano Fabrication, Shanghai Jiao Tong University, 200240 Shanghai, China; 2 Department of Micro/Nano Electronics, Shanghai Jiao Tong University, 200240 Shanghai, China; 3 Department of Neurosurgery, Center for Functional Neurosurgery, Ruijin Hospital, Shanghai Jiao Tong University School of Medicine, 200025 Shanghai, China

**Keywords:** Sensors, Nanobiotechnology

## Abstract

Implantable brain–computer interface (BCI) devices are an effective tool to decipher fundamental brain mechanisms and treat neural diseases. However, traditional neural implants with rigid or bulky cross-sections cause trauma and decrease the quality of the neuronal signal. Here, we propose a MEMS-fabricated flexible interface device for BCI applications. The microdevice with a thin film substrate can be readily reduced to submicron scale for low-invasive implantation. An elaborate silicon shuttle with an improved structure is designed to reliably implant the flexible device into brain tissue. The flexible substrate is temporarily bonded to the silicon shuttle by polyethylene glycol. On the flexible substrate, eight electrodes with different diameters are distributed evenly for local field potential and neural spike recording, both of which are modified by Pt-black to enhance the charge storage capacity and reduce the impedance. The mechanical and electrochemical characteristics of this interface were investigated in vitro. In vivo, the small cross-section of the device promises reduced trauma, and the neuronal signals can still be recorded one month after implantation, demonstrating the promise of this kind of flexible BCI device as a low-invasive tool for brain–computer communication.

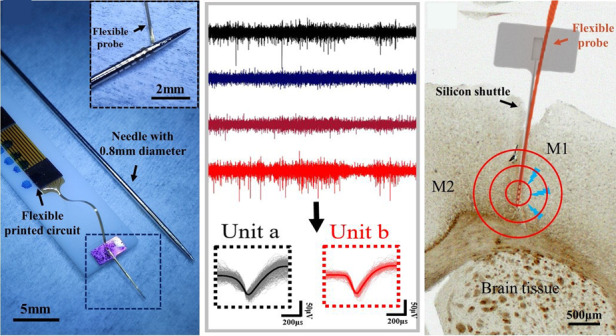

## Introduction

BCI devices, such as metal microwires or neural probes, are essential miniaturized tools to record neural signals with both single-cell and submillisecond resolution, providing critical information to understand neural circuitry. They can potentially be applied to the treatment of neurological disorders^1,2^ and brain–computer interfaces^3^ or neuroprosthetics^4^. In neuroscience research, BCI devices facilitate the understanding of sophisticated brain networks^5^. In recent decades, elaborate BCI devices, such as Utah electrodes and Michigan probes, with sophisticated structures have been achieved through the development of microelectromechanical system (MEMS) technology^6^. However, metal or silicon-based interfaces have a much higher elastic modulus than biological tissue, resulting in high bending stiffness and causing intense stress between the device and tissues^7^. Neural probes with a bulky shank or large cross-section area cause acute neuron damage and disrupt blood vessels during the process of implantation. Additionally, the relative shear motion between the device and tissue causes reoccurring cellular and vascular damage and generates various chronic immune responses^8,9^. Glial scar formation near the implants increases contact resistance and deteriorates the signal^10,11^.

In recent years, flexible BCI devices made from polymers with a smaller cross-section and bending stiffness have been manifested to be more compliant with biological tissue to reduce relative shear motion, suppress the neuroinflammatory response and improve the tissue–electrode interface^12,13^. Srikantharajah et al. proposed a minimally‑invasive flexible intracortical probe with a 250 μm^2^ cross-section per electrode, which improves the immune acceptance of the implants^14^. Musk et al. invented flexible polyimide neural threads that can be efficiently implanted into the brain by a robotic machine^15^. In addition to the modulus of the substrate, the structure of the device plays an important role in reducing the bending stiffness of the implants. Many groups have fabricated implants with ultrasmall cross-sections made by SU-8 due to its large range of possible thicknesses and mature processing technology. Luan et al. decreased the cross-section of a device to 3 × 1.5 μm^2^, whose footprints were reduced to subcellular dimensions^16^. Yang et al. presented a bioinspired design for an implantable BCI device where the key building blocks mimic the subcellular structural features and mechanical properties of neurons^17^. The bending stiffness of these ultraflexible neural implants with micrometer dimensions is comparable to the axon of a neuron, which allows a seamless interface with the brain. However, the ultrasmall cross-section fabricated by advanced processes such as electron beam lithography is costly, and the biocompatibility of SU-8 is still under debate for its mild reactivity and cytotoxicity^18^.

Polyimide (PI) is another polymer that is widely used in biomedical applications for its chemical stability and thermal resistance. In particular, photosensitive PI is suitable for photolithography processes that allow complex structures to be patterned on it, and many applications of PI implants have been explored^19–21^. The biocompatibility of commercially available PIs, such as Durimide, made by Fujifilm, has been demonstrated both in vitro and in vivo^22–24^. In this paper, we present a MEMS-fabricated flexible neural implant with an ultrathin PI substrate for low-invasive implantation. The neural implant device with a high aspect ratio and small cross-section has a low bending stiffness to suppress inflammatory responses and causes little trauma. Electrodes with two diameters on the substrate modified with Pt-black (Ptb) are designed for local field potential (LFP) and spike recording. Furthermore, a rigid silicon shuttle with an optimized structure and surface microstructure ensures the implantation accuracy of the ultrathin BCI device.

## Results and discussion

### Design of the flexible BCI device

**Fig. 1:**
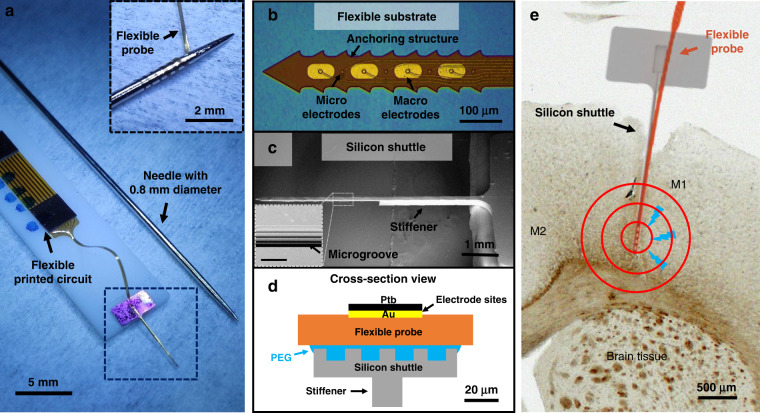
Schematic diagram of the assembled BCI device. **a** The assembled BCI device compared with a needle 0.8 mm in diameter. **b** A microphotograph of the ultrathin flexible substrate and (**c**) a SEM image of the silicon shuttle with a microgroove structure on the surface and a stiffener on the backside. The scale bar of the inset image is 100 μm. **d** The cross-sectional view of the shuttle bonded with the flexible BCI device by PEG. **e** Schematic of the flexible BCI device implanted into the mouse brain

It has been proven that devices with low bending stiffness mitigate the effect of the immune response and enable a more stable electrode–tissue interface^25^. For an implant with a square cross-section, bending stiffness K can be calculated as^26^:

1 $${{{{K}}}} = {{{{E}}}} \cdot {{{{I}}}} = ^{E \cdot b \cdot h^3/12}$$

where *E* is Young’s modulus, *I* is the rotary inertia, *b* is the width of the section and *h* is the thickness of the section. According to formula (1), the bending stiffness of the device is proportional to the modulus, width and cubic thickness. To ensure the long-term biocompatibility of the implants, photosensitive PI (Durimide 7505, Fujifilm, Japan) was used as the flexible substrate. Different spin coating speeds and methyl-2-pyrrolidinone (MNP) (87421E, Adamas, Switzerland) were applied to the polyimide precursor to acquire an ultrathin film with a thickness between nanometers and micrometers. The assembled BCI device is presented in Fig. 1a, and it can easily be wrapped around the tip of a needle 0.8 mm in diameter. On the flexible substrate (Fig. 1b), four macroelectrodes (6000 μm^2^) with an interval of 300 μm for LFP recording and another four microelectrodes with 20 μm in diameter for spike recording are evenly distributed on the tip of the PI substrate. The anchoring structures are designed on two sides of the flexible substrate to restrict further movement after the device arrives at its position^27^.

A rigid silicon shuttle is designed to achieve the implantation accuracy of the flexible BCI. The critical force of the shuttle can be calculated as^28^

2 $${{{{F}}}} = \frac{{\pi ^2EI}}{{(KL)^2}} = \frac{{\pi ^2Ebh^3}}{{12 \cdot (KL)^2}}$$

where *L* is the unsupported length and *K* is the length factor. To increase the critical force, a stiffener is designed on the backside of the silicon shuttle (Fig. 1c). Note that the T structure composed of the stiffener and the shuttle increases *I* but has no influence on the implanted cross-section area. Such a design for the shuttle promises a stable implantation procedure with minimal damage. To ensure fixation between the flexible substrate and the silicon shuttle during implantation, polyethylene glycol (PEG) is applied to temporarily bond the flexible substrate to the tip of the shuttle. The tested dissolution times of PEG with different molecular weights in PBS are listed in Table S1. Considering the insertion speed and sufficient operating time, PEG with a molecular weight of 8000 g/mol is selected. As the cross-section view shows in Fig. 1d and Fig. S1a capillary consisting of four microgrooves with 20 μm width is designed on the surface of the shuttle, and a 1-μm-thick SiO_2_ layer is deposited onto the surface of the shuttle shank to decrease the PEG contact angle. Under the capillary force, molten PEG automatically fills the whole shank of the shuttle and ensures tight coupling between the device and silicon shuttle^29^. As shown in Fig. S2, the hydrophilic surface with a capillary structure enables the PEG droplets to spread along the whole probe. With the help of the silicon shuttle, the flexible BCI device can easily penetrate the cortex of the mouse (Fig. 1e).

### Fabrication and assembly of the flexible BCI device

**Fig. 2:**
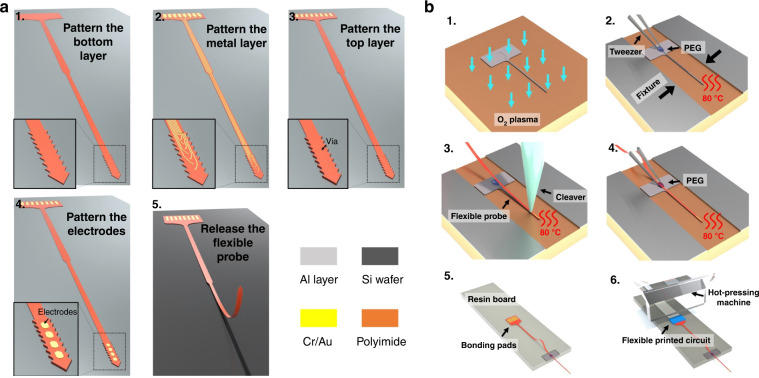
The fabrication and assembly process of the flexible substrate and silicon shuttle. **a** The fabrication process of the flexible substrate: (1) The bottom PI layer is patterned. (2) The Cr/Au layer is deposited and patterned. (3) The top PI layer is patterned. (4) The metal layer of the electrodes is patterned. (5) The flexible substrate is released in hydrochloric acid solution. **b** The assembly processes of the BCI device: (1) Shuttle is cleaned by oxygen plasma. (2) PEG is placed onto the shank of the silicon shuttle and heated to a molten state. (3) The flexible substrate is transferred to the silicon shuttle. (4) A pellet of PEG is added to the end of the device to strengthen the bonding between the flexible substrate and the shuttle. (5) The device is cooled to room temperature and then glued onto a 3D-printed resin board. (6) A flexible printed circuit is connected to the flexible device by an anisotropic conductive film

The flexible substrate and the silicon shuttle are fabricated by the MEMS process separately and then assembled together. Photosensitive polyimide (Durimide 7505, Fujifilm, Japan) is spun cast at speeds of 3000 rpm and 1500 rpm to form 2.5-μm-thick and 5-μm-thick PI films, respectively. Here, MNPs are added to the PI precursor with different mass ratios to form thinner substrates as needed, and the preparation steps of the precursor are shown in Fig. S3 and in the supplementary experimental section. As shown in Figs. 2a and S4, briefly, the bottom PI layer and metal layer are sequentially patterned on the wafer. The metal layers contain 20-nm chromium (Cr) and 300-nm gold (Au), which are used as conducting layers. For the convenience of bench tests in vitro, substrates with total thicknesses of 5 μm and 10 μm are fabricated. The nanoscale-thickness PI film is patterned only for the profilometer test. Then, the silicon shuttle is fabricated by a photolithographic process and deep reactive ion etching (DRIE), as shown in Fig. S5. Before assembly, Ptb coatings are electroplated on the electrodes to enhance the performance of the electrodes^30^. The assembly process is shown in Fig. 2b. The silicon shuttle is first cleaned by oxygen plasma and fixed onto a hotplate at 80 °C. Then, a pellet of PEG with a molecular weight of 8000 g/mol is placed onto the shank of the silicon shuttle. The melted PEG fills the microgroove by capillary action. Then, the flexible substrate is picked up by the cleaver of the wire bonding machine and transferred to the surface of the silicon shuttle. Self-alignment and close bonding are achieved between the flexible substrate and shuttle because of the capillary force. After the PEG completely fills the interface between the flexible substrate and the silicon shuttle, the PEG is cooled to room temperature for solidification. Finally, the silicon shuttle is glued onto a 3D-printed resin board, and a flexible printed circuit (FPC) is connected to the BCI device by an anisotropic conductive film (AC2056R, HITACHI, Japan).

### Mechanical properties of the flexible substrate

**Fig. 3:**
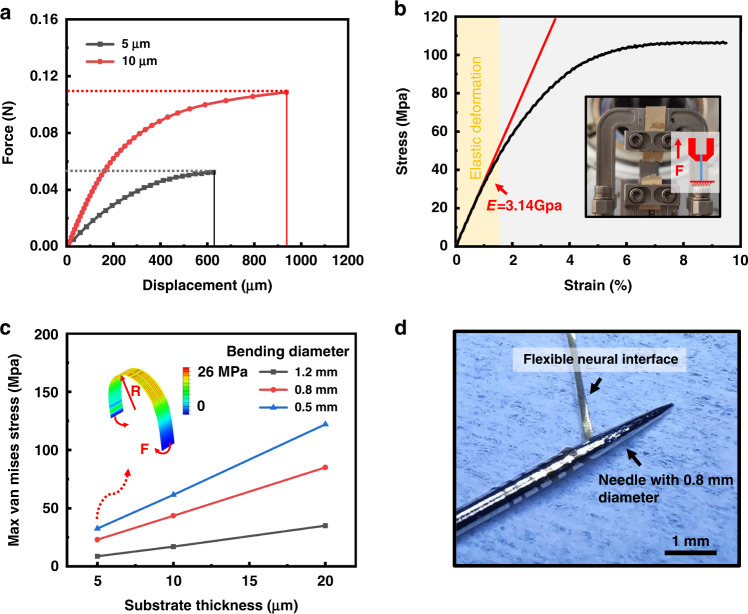
Mechanical properties of the flexible substrate. **a** The tensile force-displacement curves of the polyimide substrate with different thicknesses tested by the thermomechanical analysis system. **b** The calculated stress‒strain curve and modulus of the flexible substrate. **c** Bending analyses of the flexible substrate with different thicknesses by ABAQUS. **d** The PI substrate with 5 μm thickness twining around a needle tip with 0.8 mm diameter by water capillarity as the adhesion force

The thickness of the flexible substrate has a significant impact on the tensile strength and bending stiffness of the device. The stretching tests of devices with different thicknesses are investigated by the dynamic thermomechanical analysis system shown in Fig. 3a, and the stress‒strain curves are calculated and shown in Fig. 3b. For the substrates with thicknesses of 5 μm and 10 μm, the maximum force before fracture is 52 mN and 107 mN, respectively. As calculated in the elastic deformation segments in linear range, the Young’s modulus of the fabricated device is 3.14 GPa. The thicker substrate has a higher fracture force, which ensures the stability of the interface when implanted into the tissue or in the micromovement of the tissue. To evaluate the flexibility of the PI substrate in different thicknesses, bending diameters of 1.2 mm, 0.8 mm and 0.5 mm are applied to the flexible probe, and the maximum van Mises stresses are calculated in the finite element analysis (FEA) software ABAQUS (SIMULIA, Pawtucket, RI, USA) in Fig. 3c and Fig. S6. The device with the thinner substrate is more bendable than that with the thicker substrate, which leads to smaller stress and causes less tissue damage. As the results show, the trade-off between the fracture force and the bending stiffness should be carefully considered in practical applications by controlling the thickness of the substrate. The PI substrate with a thickness of 5 μm has low bending stiffness, which allows its wrapping around the 0.8 mm needle tip by water capillarity, as shown in Fig. 3d.

### Thickness scalability of the flexible substrate

**Fig. 4:**
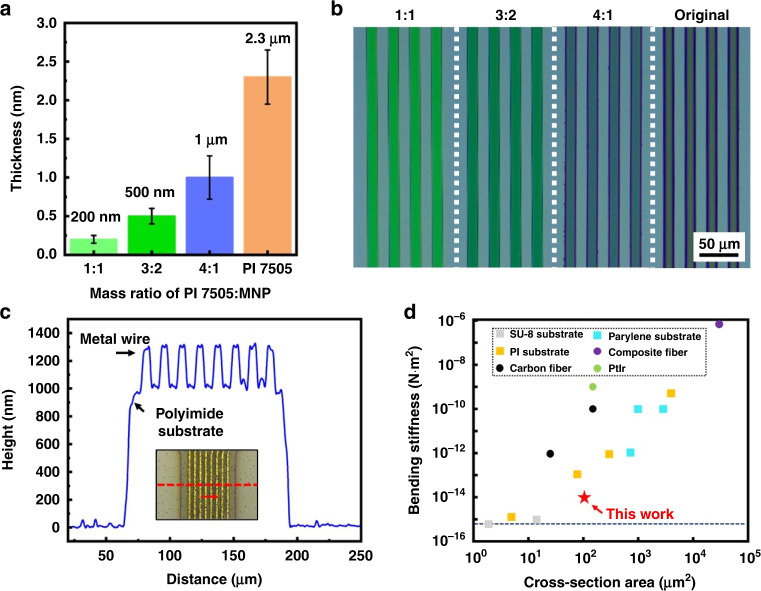
The thickness scalability of the polyimide substrate. **a** The nanoscale substrate fabricated by the PI precursor mixed with MNPs with different mass ratios. **b** Micrograph of patterned PI films of different thicknesses formed by mixing the PI precursor and MNPs. **c** The thickness tested by the profilometer of the 1-μm-thick PI substrate. **d** Bending stiffness of the flexible implants in recent studies

A thinner substrate helps to improve the bending properties of the flexible BCI device and reduce tissue damage in chronic implantation. As described in the fabrication section, by adding MNP to the PI precursor, 200 nm-, 500 nm- and 1-μm-thick PI films are fabricated and tested by a profilometer (Dektak XTL, Bruker, Germany), as shown in Fig. 4a, while the original PI layer is 2.3 μm thick. The iminated and patterned PI films are shown on the right side of Fig. 4b. Compared with the PI at the microscale, the PI at the nanoscale shows a significant color change caused by the thin-film interference. Additionally, the uniform color formed by the same mass ratio illustrates the uniformity of the film thickness. However, increasing the biocompatibility of the device by decreasing its cross-section comes at the expense of the tensile strength of the device. In addition, for materials with the same water permeability, the thinner substrate allows body fluids to permeate the interior of the device more quickly, which is not conducive to long-term implantation. Considering both the mechanical and electrochemical characteristics of the implants, 500-nm-thick PI in a single layer is applied to fabricate the substrate of the BCI device, and a total 1-μm-thick device is measured by the profilometer for the whole device in Fig. 4c. According to Formula (1), the bending stiffness of the 1 μm-thick device is 2.6 × 10^−14^ N m^2^, which is smaller than most polymer implants summarized in Fig. 4d and Table S2 and close to the bending stiffness of axons^14,16,17,31–37^.

### Mechanical properties of the silicon shuttle

**Fig. 5:**
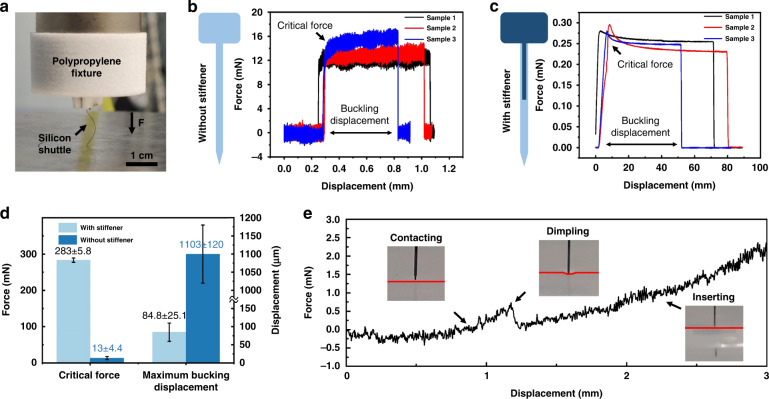
Mechanical properties of the silicon shuttle. **a** The configuration of the buckling test. **b** Typical displacement curves of the silicon shuttle without a stiffener. **c** Typical displacement curves of the silicon shuttle with a stiffener. **d** Statistical results of the critical force and buckling displacement for two different shuttles in (**b**) and (**c**). Error bars show SD, *n* = 8. **e** The force-displacement curves when the shuttle inserts into the agar model

To accurately deliver the flexible BCI device into the cortex, a silicon shuttle is fabricated by a silicon-based MEMS process. According to Formula (2), the critical force is proportional to the bending stiffness of the shuttle. Once the stress exceeds the critical force and the shuttle is in a buckling state, it is unstable and can be broken. To strengthen the critical force of the shuttle, a stiffener is designed on the backside of the shuttle, and the critical force of the optimized design is tested by a universal testing machine. Figure 5a shows the mechanical test configuration. The force is applied uniaxially from the polypropylene fixture to obtain the force-displacement curves of the shuttle. Before the mechanical test of the shuttle, the shuttle with a T-shaped stiffener is analyzed with ABAQUS. Figure S7 shows the Mises stress distribution of the traditional structured silicon shuttle (without a stiffener) and the improved shuttle under the same compressed distance. The maximum stress distribution in the silicon shuttle with/without stiffener is 399 MPa and 312 MPa, respectively; thus, the stiffener avoids the unstable bending of the silicon shuttle under compression and provides a concentrated force. The typical force-displacement curves of the shuttle with these two structures can be found in Fig. 5b, c, and the critical force (the peak value of the force-displacement curves) and buckling displacement (the part of the displacement where the stress is not zero) are recorded and summarized in Fig. 5d. The results show that the shuttle with the stiffener has a larger critical force and smaller buckling displacement, and the average critical force of the shuttle with the stiffener is 287.2 ± 5.8 mN (mean ± standard error), which is 20 times larger than the original value. A large critical force and small displacement are important for the shuttle, which means that the shuttle can penetrate harder tissue and does not deflect during implantation. On the other hand, the agar model with a concentration of 0.6% in DI water is used for the insertion experiments in Fig. 5e. The maximum insertion force (0.8 ± 0.4 mN) appears when there is a dimple on the surface. After the shuttle penetrates the surface, the insert force suddenly drops and then gradually increases with the implantation depth because of the increase in sliding friction. The critical force of the optimized shuttle far exceeds the maximum insertion force, which promises a stable implantation process of the flexible substrate.

### Electrochemical modification of the microelectrodes

**Fig. 6:**
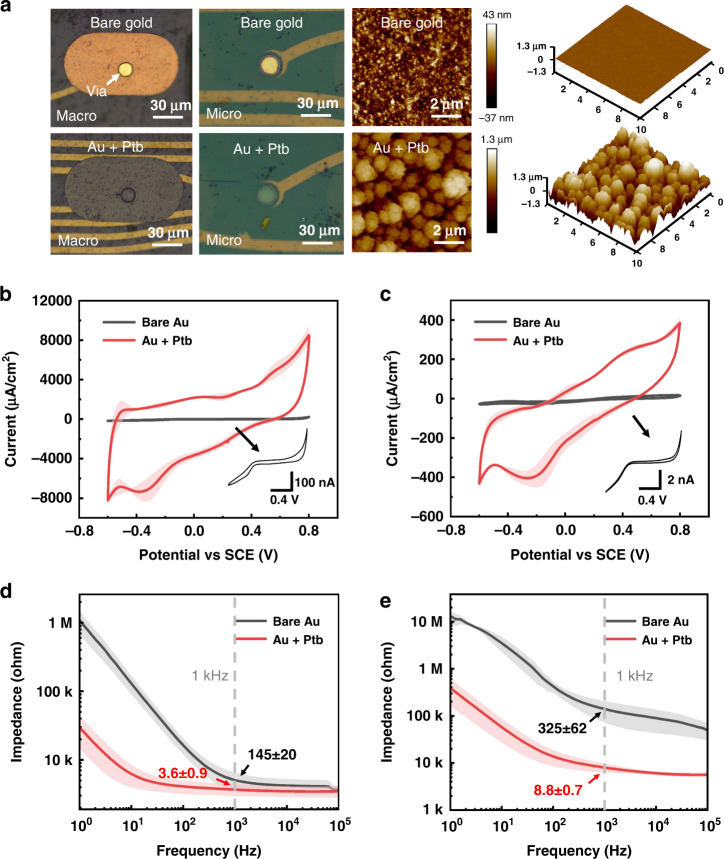
Electrochemical characterization of the electrode sites. **a** Microscope and AFM images of surface morphology before and after Ptb modification. Comparison of CV before and after Ptb modification for the (**b**) macroelectrodes and (**c**) microelectrodes. Comparison of EIS before and after Ptb modification for the (**d**) macroelectrodes and (**e**) microelectrodes. Error band shows SD, *n* = 10

The performance of electrophysiological signal recording highly depends on the electrochemical properties of the electrode sites. Therefore, recording sites are modified with Ptb to improve their performance. The cyclic voltammetry (CV) and impedance spectrum (EIS) are measured after electroplating and compared with the original spectrum. As shown in Fig. 6a, for both macro- and microelectrode sites, Ptb is uniformly coated on Au sites. After Ptb plating, the surface morphology was measured by atomic force microscopy (AFM), and the roughness of the electrode surface increased from 11 ± 1 to 310 ± 7.2 nm. The cauliflower-shaped particles of Ptb significantly increase the effective surface area of the electrode sites, which helps to decrease the impedance. After Ptb is electroplated by repetitive current pulses, the typical CV and EIS comparison diagrams were recorded and are shown in Fig. 6b–e. Compared to the Au electrode sites, the sites modified with Ptb not only have a larger effective surface area but also exhibit pseudocapacitance characteristics, which results in a significant increase in the CV area and a decrease in impedance across the spectrum. After Ptb modification, the charge storage capacity (CSC) increases from 2.9 ± 0.6 to 55.6 ± 14.5 mC/cm^2^ for macroelectrode sites and from 1 ± 0.2 to 24.3 ± 6.5 mC/cm^2^ for microelectrode sites. The measured impedance at 1 kHz decreases to 8.8 ± 0.7 kΩ and 3.6 ± 0.9 kΩ for micro- and macro-electrode sites, respectively. The recording sites with lower impedance and larger CSC help to reduce baseline noise and improve neural recording.

### Electrophysiological recordings from the cortex of the mouse

**Fig. 7:**
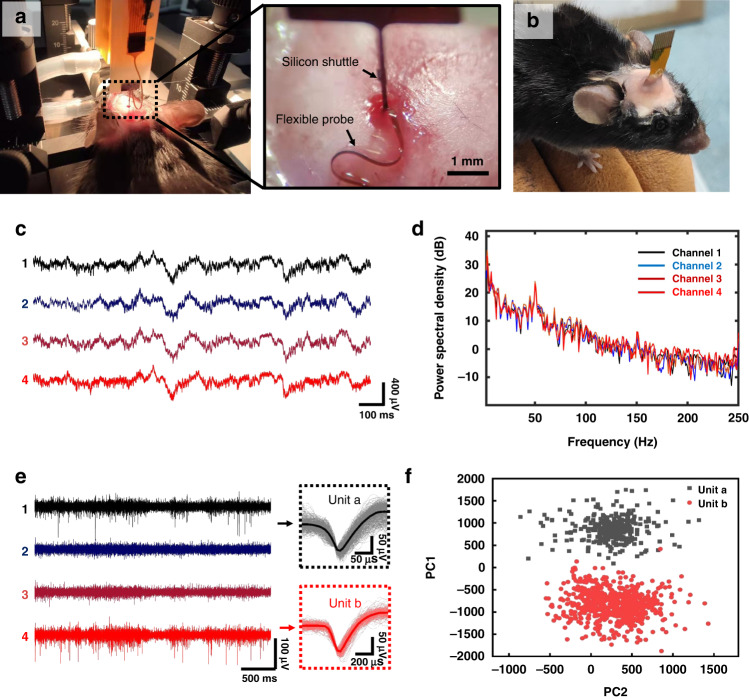
Neural recording in vivo. **a** The BCI device is implanted into the mouse brain. **b** The flexible BCI device was fixed by dental cement after implantation. **c** LFP recorded by four macroelectrode sites. **d** PSD of the LFP recorded by the macroelectrode sites. **e** The spontaneous waveform and single-unit activities recorded by the four microelectrode sites after high-pass filtering. **f** PCA of the two recorded single-unit activities. Each point represents component 1 (PC1) and component 2 (PC2) of a single spike

The flexible BCI device is implanted into the M1 of the mouse by the silicon shuttle (Fig. 7a) according to the procedure shown in Fig. S8. As shown in Fig. 7b, implants with light weight and small pads allow free movement of the animal, which is beneficial for long-term recording. Spikes and LFP were recorded successfully 30 days after implantation, and all neural activities were recorded by a digital data acquisition system (Intan Technology). Figure 7c shows the LFP recorded by the macroelectrode sites. The power spectra density (PSD) is calculated in Fig. 7d. The recorded intracortical LFP exhibits a larger high-frequency power spectrum than the ECoG electrodes did in our previous study^38,39^, which shows the advantages of these invasive electrodes. Figure 7e shows the spontaneous waveform recorded by the four microelectrodes after high-pass filtering by a 4-pole Bessel digital filter in an offline sorter with a cutoff frequency of 250 Hz. Two single-unit activities from channel 1 and channel 4 are detected, and two distinct clusters of these two units are sorted by PCA in Fig. 7f. The clear spike waveform demonstrates the recording ability of the microelectrode sites. We then investigate the impedance variation of the electrodes, as shown in Fig. S9. For both kinds of electrodes, the impedance generally increases because of the proliferation of gliocytes around the interface^40–42^. However, inflammatory reactions and gliosis are found to be reduced in long-term application of the flexible interface compared to the rigid interface^43,44^.

### Histological analysis of the immune response

**Fig. 8:**
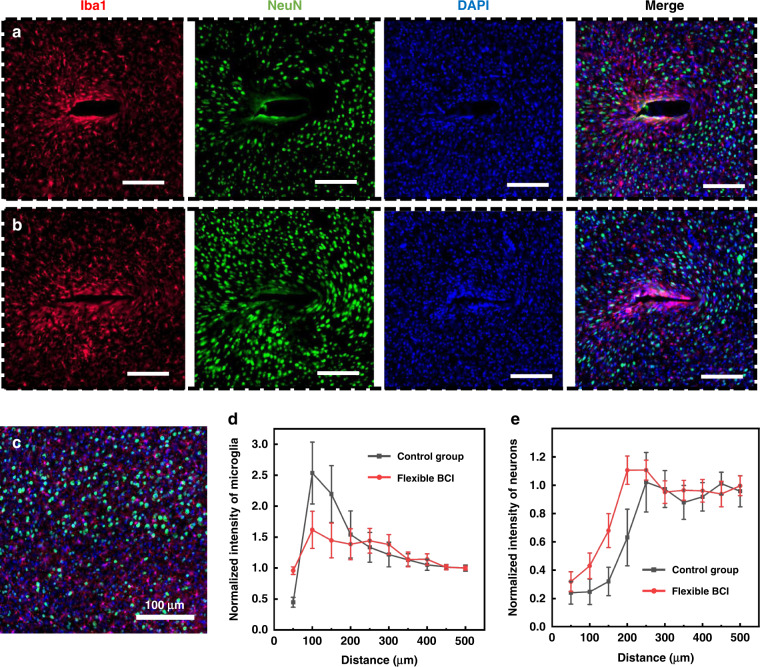
Histological studies of tissue response to the implanted flexible BCI device. Immunofluorescence images of tissue responses to a traditional neural probe 30 μm in thickness (**a**) and the flexible BCI device with a 5-μm-thick substrate (**b**), labeled for microglial (Iba1, red), neurons (NeuN, green) and nuclei (DAPI, blue); scale bars show 100 μm. The merged fluorescence image of the normal tissue (**c**) and the normalized fluorescence intensities of microglia (**d**) and neurons (**e**) against background values plotted as a function of distance from the center of the electrode footprint. Error bars show SD, *n* = 5

To evaluate the biocompatibility of the flexible BCI device in vivo, we performed postmortem histology 30 days after implantation. A control probe (30 μm in thickness) with the same width in the contralateral M1 region was used for comparison. Tissue sections were stained by immunohistochemistry for markers chosen to visualize the presence of microglia (Iba1), neuronal nuclei (NeuN) and cell nuclei (DAPI). The results of brain slice fluorescence microscopy images implanted with the control group and the flexible BCI device are shown in Fig. 8a, b, respectively. For the implants in the control group, there is major microglia accumulation at the device−tissue interface, which forms a tighter sheath encapsulating the implant. The dense gliocytes that encapsulate the implants and isolate them from the neurons are one of the main reasons for reduced performance of electrodes. Meanwhile, 100–200 μm around the implantation site appears to be a clear neuron “kill zone” at four weeks after implantation, which is characteristic of the brain tissue inflammatory response to implanted devices^10,45^. For the ultrathin BCI device, benefiting from its small footprint and low bending stiffness, less glial encapsulation and a higher density of neurons are found around the implantation site, and a smaller tissue cavity is caused by the device. At the same time, the fact that the neurons are alive suggests low cytotoxicity of the PI substrate we selected within 30 days. Figure 8c shows the merged micrograph of normal tissue, which is used as the calculated background of the fluorescence intensity. The normalized fluorescence intensity profiles of microglia and neurons are plotted as a function of distance from the center of implantation, as shown in Fig. 8d, e, which quantitatively reflects the inflammatory response caused by the implants. As the fluorescent immunostaining results show, a thinner substrate causing little injury to the tissue results in a lesser immunological reaction and promises a longer lifetime.

## Conclusion

In summary, we have developed a flexible BCI device for low-invasive neural recording. The BCI contains a PI substrate with ultrathin thickness and a silicon shuttle designed for accurate placement. The robust batch MEMS fabrication process promises a controlled size design of the device. We systematically investigated both the mechanical and electrochemical characteristics of the ultrathin PI substrate and the silicon shuttle in vitro. Because of the low invasive footprint and low bending stiffness, LFP and single-unit activities are successfully recorded by different electrodes after implantation. The immunohistochemical staining results also show reduced neuronal cell loss and reduced immune reactions caused by the thinner substrate. Even so, the cross-section of the implants should be carefully considered for its mechanical robustness and electrochemical stability in practical applications. Moreover, this kind of flexible BCI device can still be further improved by reducing its width or adopting the mesh structure in the future.

## Materials and methods

### Fabrication process of the flexible substrate

The flexible substrate is fabricated using the standard MEMS fabrication process, which is shown in Figure S4 with the following steps. First, methyl-2-pyrrolidinone (87421E, Adamas, Switzerland) was added to the photosensitive polyimide (Durimide 7505, Fujifilm, Japan) with different mass ratios to form PI films with different thicknesses as the premachined substrate material. After the reagents were fully mixed by the mixer, the air bubble in the reagent was removed from the vacuum chamber. Then, the flexible MEMS process was used to fabricate the flexible neural implant. Rinsed by the standard cleaning method, the wafer was evaporated with 400-nm-thick aluminum as the sacrificial layer. The PI reagent was spin-casted (speed, 3000 rpm) onto the wafer to form a nanoscale film. After soft baking, photolithography, development in HTRD2 (Fujifilm, Japan) and rinsing in RER600 (Fujifilm, Japan), the patterned PI was finally cured in N_2_ at 300 °C for 1 h to realize imidization. The 20-nm chromium (Cr) and 300-nm gold (Au) were deposited onto PI by a plasma sputtering system and patterned by an ion beam etching system (LKJ 150, Advanced Ion Beam Ltd., China). Then, another PI film was spin-coated and patterned by photolithography to form the top layer of the flexible substrate. To form the macro electrode sites, the second metal (Cr/Au 20/300 nm) layer was sputtered onto the substrate and patterned by ion etching. Finally, the wafer was immersed into hydrochloric acid solution (HCl:H_2_O = 1:5) to release the device.

### Fabrication process of the silicon shuttle

The silicon shuttle was fabricated using the standard silicon MEMS fabrication process, which is shown in Figure S5 with the following steps. The silicon wafer was rinsed by the standard cleaning method. A 5-μm-thick photoresist (PR) was spin-coated onto the wafer and patterned by photolithography. Deep reactive ion etching (DRIE) was used to form the microgrooves and the reservoir. Then, 200-nm-thick silicon nitride and 800-nm-thick silicon oxide were deposited on two sides of the wafer by a plasma-enhanced chemical vapor deposition system. The silicon nitride layer has a tensile stress that compensates for the compressive stress from the oxide silicon. After spin-coating the 15-μm-thick PR onto the front side of the wafer, the second lithography step was used to generate front release lines. Reactive ion etching (RIE) and DRIE were used to remove the oxide layer and form the profile of the silicon shuttle, respectively. Then, another layer of PR was spin-coated on the front side of the wafer to protect the structures during back etching. The PR was patterned on the back-side of the wafer, and one thin strip of PR, slightly narrower than the shank, was reserved to form the reinforcement of the probe. The 1-μm-thick oxide layer and 370-μm-thick silicon layer were then etched by the back RIE and DRIE, respectively. Finally, the wafer was put into acetone to remove the PR and release the shuttles from the substrate.

### Modification of electrode sites

The electrode sites were modified with Ptb to improve the electrochemical performance. The Ptb coatings were electroplated by applying repetitive current pulses (duty ratio of 5 ms:500 ms, peak current density of 4.5 A/cm^2^, and 480 cycles) in chloroplatinic acid solution (3% chloroplatinic acid and 0.01% lead acetate in deionized water) under ultrasonication. CV and EIS analyses were used to calculate the CSC and investigate the characteristic changes of electrode sites before/after Ptb was modified.

### Electrochemical characterization

The CV and electrochemical EIS were measured by an Autolab (PGSTAT204, Switzerland) in PBS (0.01 M, pH 7.4) with a scan rate of 100 mV/s, a conventional three-electrode system saturated calomel electrode (SCE) as the reference electrode, and Pt foil as the counter electrode (voltage range from −0.6 V to 0.8 V). The CSC is calculated as ${{{\mathrm{CSC}}}} = \frac{1}{{vA}}{\int}_{E_c}^{E_a} {\left| i \right|} {\rm{d}}E$, where *E* is the electrode potential, *i* is the measured current, *A* is the geometric area of the electrode site, *v* is the scan rate, and *E*_*a*_ and *E*_*c*_ are the anodic and cathodic potential limits, respectively. EIS was measured in the frequency range from 0.1 Hz to 100,000 Hz with an input voltage amplitude of 0.01 V. CV analysis was used to investigate possible charge transfer reactions and calculate the CSC of electrode sites before/after Ptb modification.

### Implantation procedure of the flexible BCI device

All animal studies and experimental procedures in this manuscript were approved by the animal care and use committee of Shanghai Jiao Tong University. The detailed implantation steps are shown in Fig. S8. First, mice were anesthetized by the intraperitoneal injection of pentobarbital (100 mg/kg) and mounted in a stereotaxic holder to adjust their skulls to be parallel with the reference panel. Then, the silicon shuttle attached to the flexible neural probe was inserted into the primary motor cortex M1 (AP: +1 mm ML: +1.5 mm DV: −1 mm) by the stereotaxic apparatus. The FPC at the end of the device was separated from the resin board, and PBS was dripped to the tip of the BCI device to dissolve the PEG. After waiting for 5 min for the PEG fully dissolved, the resin board and silicon shuttle were detached from the flexible substrate and pulled away from the tissues. Then, a stainless-steel screw on the back of the skull was used as the ground. Finally, all the implants were covered with dental cement except for the connecting pads.

## Supplementary information

Supporting Information
