# Supplementary material for: A flexible neural implant with ultrathin substrate for low-invasive brain–computer interface applications
Source: Microsyst Nanoeng. 2022 Dec 25;8:133. doi: 10.1038/s41378-022-00464-1 (PMC9789992; doi:10.1038/s41378-022-00464-1)
Supplement: Supplementary file 1 — Supporting Information [file 41378_2022_464_MOESM1_ESM.docx]

Supporting Information

**A flexible neural implant with scalable thickness for low-invasive brain-computer interface application**

Zhejun Guo^1,2^, Fang Wang^3^, Longchun Wang^1,2^, Kejun Tu^1,2^, Chunpeng Jiang^1,2^, Wen Hong^1,2^, Ye Xi^1,2^, Xiaolin Wang^1^, Bin Yang^1^, Bomin Sun^3^, Zudelin^1*^, Jingquan Liu^1*^

^1^ National Key Laboratory of Science and Technology on Micro/Nano Fabrication, Shanghai Jiao Tong University, Shanghai 200240, China.

^2^ Department of Micro/Nano Electronics, Shanghai Jiao Tong University, Shanghai 200240, China.

^3^ Department of Neurosurgery, Center for Functional Neurosurgery, Ruijin Hospital, Shanghai Jiao Tong University School of Medicine, Shanghai 200025, China.

*CORRESPONDING AUTHOR
Prof. Jingquan Liu & Dr. Zude Lin

E-mail: [jqliu@sjtu.edu.cn](mailto:jqliu@sjtu.edu.cn); linzude@sjtu.edu.cn

This file includes: Supplementary experimental section, supporting table S1-S2 and figures S1-S9.

**Supplementary experimental section:**

***Mechanical simulation of the flexible substrate:*** ABAQUS commercial software (ABAQUS Analysis User’s Manual 2010; V 6.10) was used to study the bending properties of the flexible substrate. Eight-node 3D solid elements (C3D8R) and four-node shell elements (S4R) were used to model the elastic substrate and the metal layers. Young’s moduli (E) and Poisson’s ratios (ν) were E_Cr_ = 279 GPa and ν_Cr_ = 0.21 for Cr; E_Au_ = 79 GPa and ν_Au_ = 0.42 for Au.; E_PI_ = 3.1 GPa and ν_PI_ = 0.34 for PI.

***Mechanical simulation of the silicon shuttle:*** ABAQUS commercial software (ABAQUS Analysis User’s Manual 2010; V 6.10) was used to study the mechanical properties of the silicon shuttle. The shuttle with/without stiffener was compared through the maximum strain under the same stress. Because the crystal orientation of the probe’ axial is <110>, linear elasticity was used to define the silicon, and the Young’s moduli and Poisson’s ratios were set to 169 GPa and 0.2782 respectively, and the mass density was set to be 2.33g/cm^3^. The tip of the shuttle was pinned and the end of the shuttle was given a constant pressure along the axial of the needle to obtain the buckling state. The main body of the shuttle was modeled by the hexahedron element (C3D8R) and the neck of the shuttle was modeled by quadratic tetrahedron (C3D10) to ensure accuracy.

***The precursor preparation for different thickness polyimide substrate:*** The substrates with thickness with 10 µm, 5 µm were fabricated by the Photosensitive polyimide (Durimide 7505, Fujifilm, Japan) spin casting with speeds of 1500 rpm and 3000 rpm, respectively. The substrates with thickness with 200 nm, 500 nm and 1 μm in thickness were fabricated by the mixture of polyimide (Durimide 7505, Fujifilm, Japan) precursor and MNP (87421E, Adamas, Switzerland) with the mass ratio of 1:1, 3:2 and 4:1 respectively, and then spin casting with speeds of 3000 rpm.

***Compression tests of the silicon shuttle:*** The mechanical property of the silicon shuttle was tested by universal testing machine (AGS-X10KN, SHIMADZU, Japan), and the compression mode was used in the test and the maximum load capacity was set to be 50 N. A fixture 3D printed board by polypropylene was used to clamp the shuttle. Velocity at 2 mm/min and force with 1 mN were set as the speed of load cell and the initial displacement monitoring point, respectively. To acquire the critical force, peak of the force-displacement curves in repeated trials were selected and counted. The distance between the initial displacement monitoring point to the point that the silicon shuttle was flexed and broken (i.e., the force drops to zero suddenly) was calculated as the maximum bucking displacement of the shuttle.

***Surface morphological and topographical characterization:*** The morphology of the surface was characterized using a Zeiss Ultra Plus SEM. The topographical images for the electrodes were obtained on an atomic force microscope (AFM) system (FastScan Bio) operated in noncontact mode. The scanning area was 10 × 10 µm.

***Young's modulus test of the flexible substrate:*** In order to acquire the bending stiffness of the flexible substrate, the ultrathin was tested by dynamic thermomechanical analysis system (DMA Q800, TA Instrument). The sample on one side was fixed, whereas the other side was movable and the load was uniaxially applied from one side, the force-displacement curve was plotted and the stress-strain curve was calculated, the gradient at the linearization range of the stress-strain curve was calculated as the modulus.

***LFP and spikes recording:*** After the mice awake, spontaneous neuronal activity was recorded at a sampling frequency of 20 kHz by a Stim/Recording system (RHS2116 amplifier; Intan Technology). Signals, high-pass filtered with the cut-off frequency of 250 Hz, were utilized to distinguish the local field potential (LFP) and neural spikes. Offline sorter was used to detect the spike activities and negative three times of standard deviation of recorded signals were set as thresholds for detecting spike events. Principal component analysis (PCA) of offline sorter was applied to distinguish individual spikes.

***Immunohistochemistry****:* Four weeks after the BCI device implantation, mice were anesthetized with an overdose of chloralhydrate and transcardially perfused with 0.9% saline followed by 4% paraformaldehyde (PFA) in PBS. Then, the brain was removed and postfixed in 4% PFA for 24 h at 4°C. The brain was sliced into 35-μm-thick sections perpendicular to the implantation or sectioned coronally at 50 μm thickness and stored in PBS. The sections were blocked with 5% BSA in PBS with 0.3% Triton X-100, and incubated with a goat polyclonal antibody against Iba1 (the marker of the microglia; 1:500, Cat. #ab104224, abcam) and a mouse polyclonal antibody against NeuN (the marker of the neuron; 1:1000, Cat. #ab5076, abcam) at 4°C overnight. After washing with PBS, the sections were incubated with AlexaFluor 555 donkey anti-goat IgG (1:500; Invitrogen, Immunostaining of Iba1) or AlexaFluor 647 donkey anti-mouse IgG (1:500; Invitrogen, Immunostaining of NeuN) for 2 h at room temperature. PBS-washed sections were then coverslipped with Fluoromount-G (#0200-20, SouthernViotech) to prevent quenching of fluorescence and stain cell nuclei. All the mouse brains sections were imaged using the Olympus VS200 microscope. Normalized neuron/microglia density cells were calculated with ImageJ software, circular outlines of 50 μm length were segmented up to the distant uninjured regions (500 μm in total), and the normalized fluorescence intensity (uninjured regions defined as background) for all pixels was calculated to compare with each other.

**Supplementary tables:**

**Table S1.** The dissolving time of PEG with different molecular.

| **PEG molecular**  **weighte (g/mol)** | **PEG dissolved after:** | | | |
| --- | --- | --- | --- | --- |
|  | **30s** | **1min** | **3min** | **5min** |
| **1000** | ✓ | ✓ | ✓ | ✓ |
| **2000** | 🗶 | ✓ | ✓ | ✓ |
| **6000** | 🗶 | 🗶 | ✓ | ✓ |
| **8000** | 🗶 | 🗶 | 🗶 | ✓ |
| **10000** | 🗶 | 🗶 | 🗶 | 🗶 |

✓: PEG has been dissolved; 🗶: PEG has not been dissolved completely;

**Table S2.** Comparison of the bending stiffness in recent researches.

| **Author** | **Number of electrodes** | **Cross-section (μm^2^)** | **Bending stiffness (N‧m^2^)** |
| --- | --- | --- | --- |
| Yang et al.[1] | 1 | 2×0.9 | 5.7×10^-16^ |
| Luan et al.[2] | 8 | 10×1.5 | 10^-15^ |
| Guan et al.[3] | 1 | 10×1.5/3×1.5 | 10^-15^ |
| Musk et al.[4] | 32 | 6×50 | 9×10^-13^ |
| Gao et al.[5] | 1 | 4×20 | 10^-13^ |
| Pas et al.[6] | 6 | 4×180 | 10^-12^ |
| Wang et al.[7] | 8 | 20×150 | 10^-10^ |
| Srikantharajah et al.[8] | 8 | 10×90 | 10^-10^ |
| Lu et al.[9] | 1 | 10^2^/4 25^2^/4 | 10^-12^/10^-10^ |
| Vitale et al.[10] | 1 | 25^2^/4 | 10^-9^ |
| Canales et al.[11] | 4 | 350^2^/4 | 10^-6^ |
| This work | 8 | 1×100 | 2.6×10^-14^ |

**Supplementary figures:**


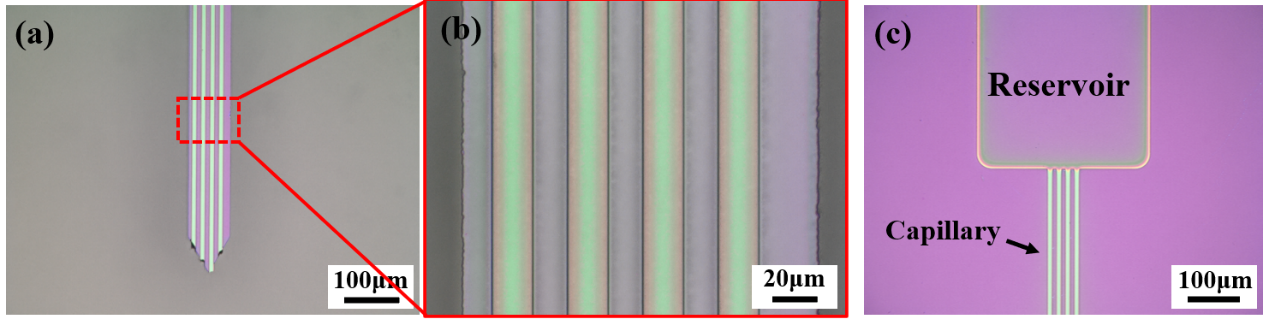


**Figure S1.** Microphotograph of the microgrooves on the silicon shuttle. (a) Microgrooves on the shank of silicon shuttle. (b) Enlargement of multi-slot structure. (c) The PEG reservoir connected with the microgrooves on the tab of the shuttle.


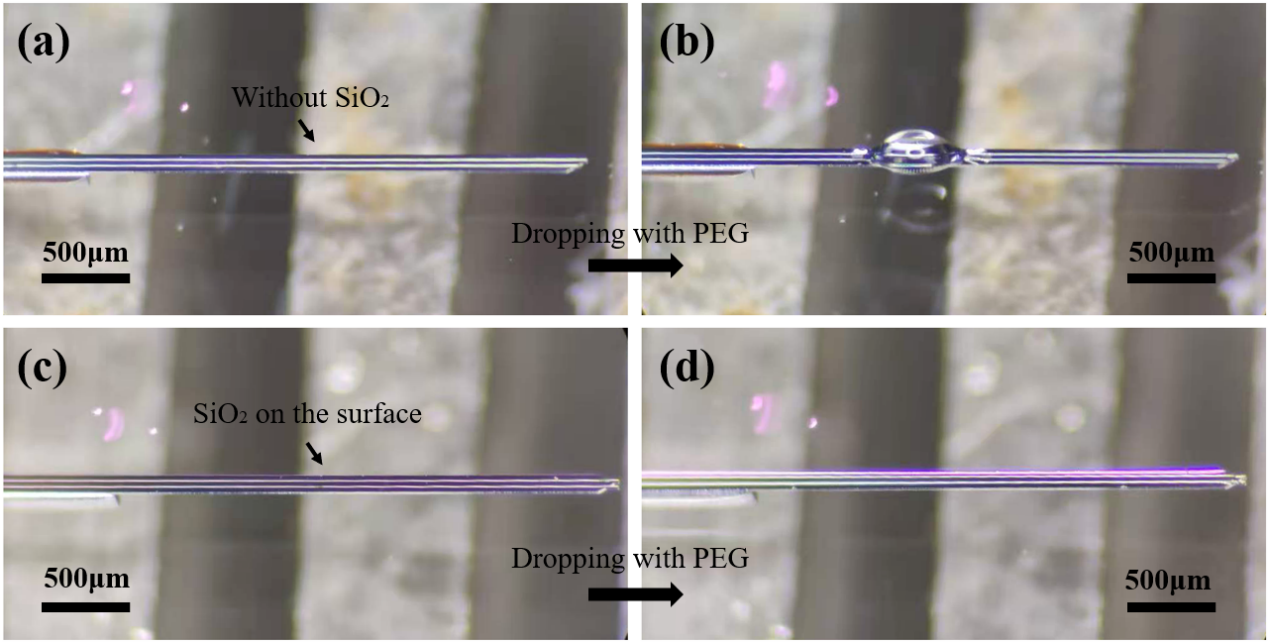


**Figure S2.** PEG wettability on silicon shuttle. (a) There is no SiO_2_ deposited on the surface of the shuttle and (b) the PEG spread out on the shank with difficulty. (c) The SiO_2_ deposited on the surface of the shuttle and (d) the PEG spread out homogeneously on the silicon shank.


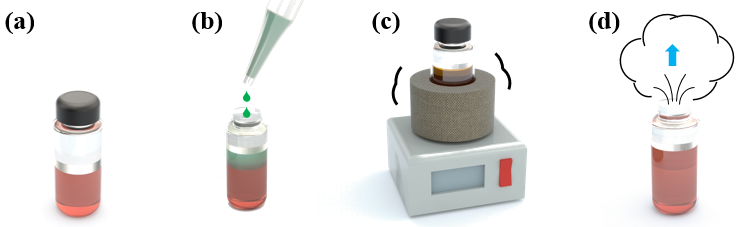


**Figure S3.** Preparation processes of the nanoscale polyimide precursor. (a) Preparation the photosensitive polyimide. (b) Adding the MNP to the precursor. (c) The reagent is mixed by the mixer. (d) Air bubbles removing in the vacuum chamber.


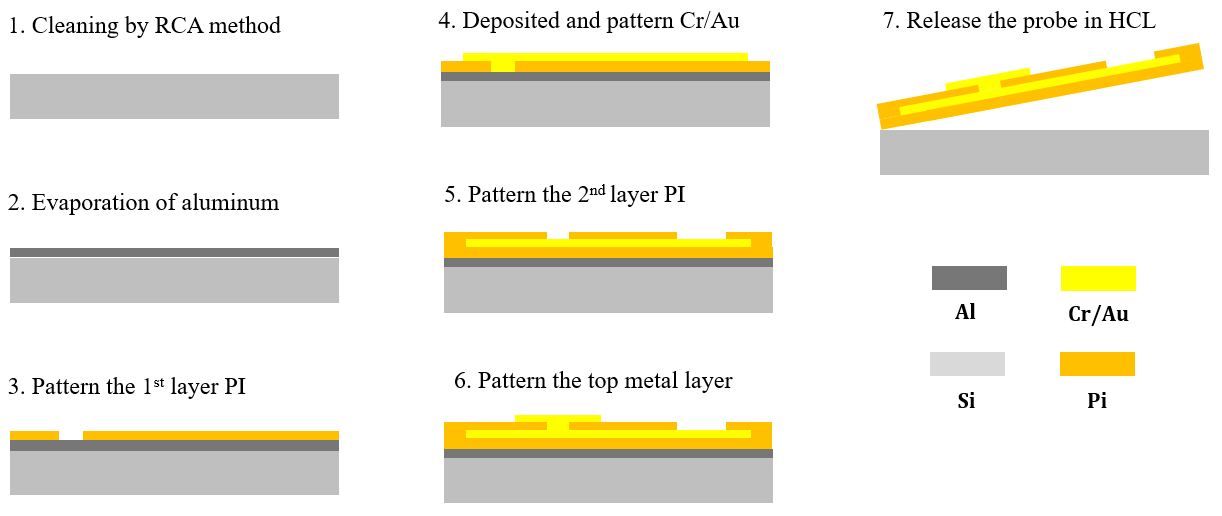


**Figure S4.** Fabrication processes of the flexible BCI device.


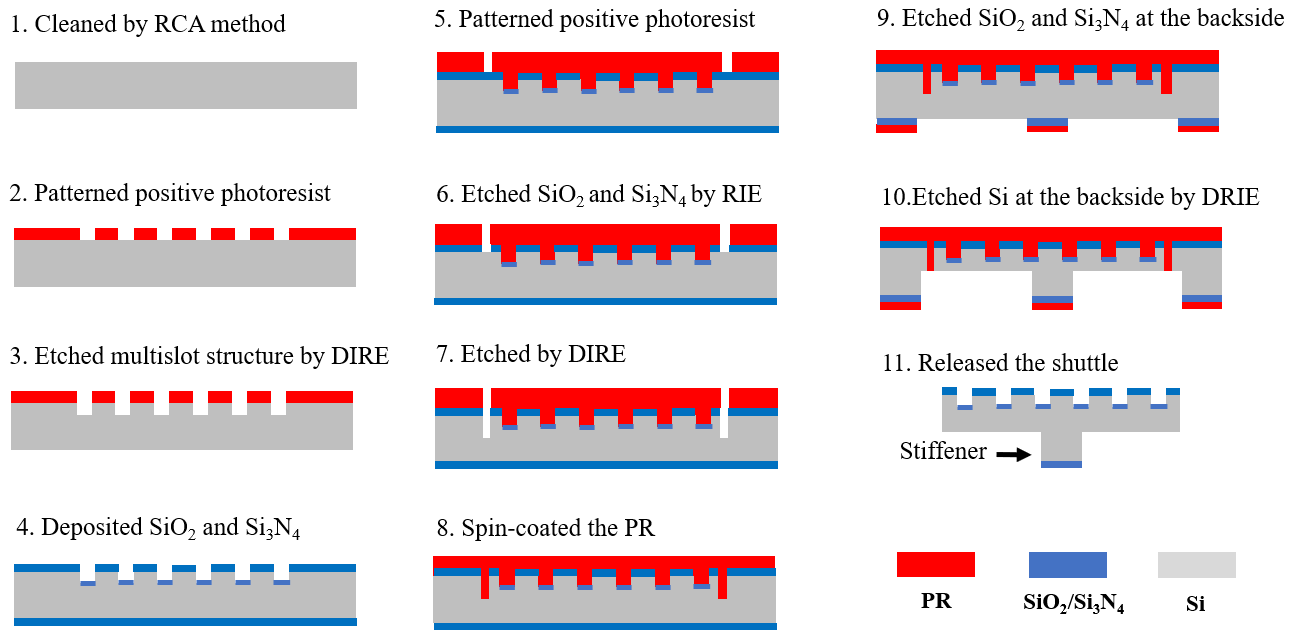


**Figure S5** Fabrication processes of the silicon shuttle.


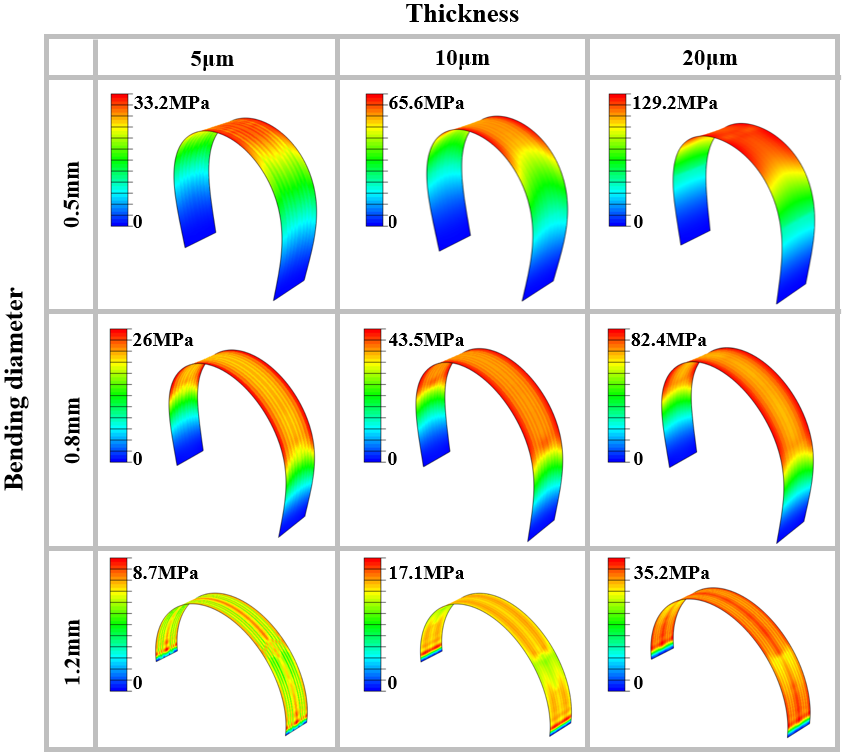


**Figure S6.** Van Mises stresses distribution of the PI substrate in different thicknesses and bending diameter.


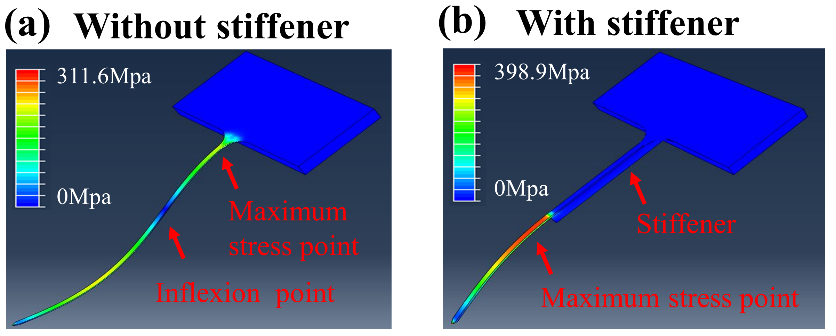


**Figure S7.** The Mises stress distribution of the silicon shuttle under compression with different structures in simulation. (a) The shuttle without stiffener. (b) The shuttle with stiffener.


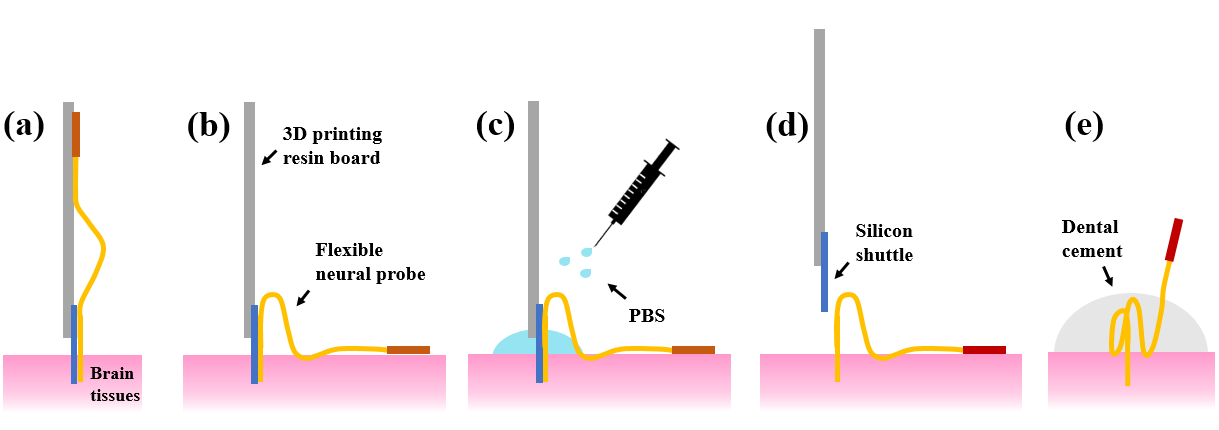


**Figure S8.** Implantation procedure. (a) Inserting the assembled device into the brain tissues by micromanipulator. (b) Separating the pad of the flexible substrate from the 3D printing resin board. (c) PBS was dripped to the tip between the device and the silicon shuttle to solve the PEG. (d) The resin board and silicon shuttle were detached from the flexible substrate and pulled away from the tissues. (e) Dental cement was used to fix the flexible BCI device on the brain tissues.

**
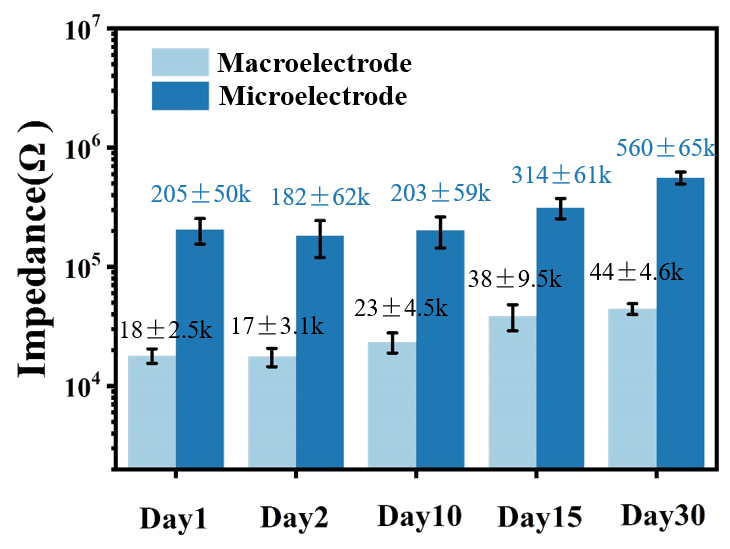
**

**Figure S9.** The variation of impedance at 1 kHz in 30 days in vivo.

**References:**

1. Yang, X. et al. Bioinspired neuron-like electronics. *NAT MATER* **18**, 510-517 (2019).

2. Luan, L. et al. Ultraflexible nanoelectronic probes form reliable, glial scar-free neural integration. *SCI ADV* **3**, e1601966 (2017).

3. Guan, S. et al. Elastocapillary self-assembled neurotassels for stable neural activity recordings. *SCI ADV* **5**, v2842 (2019).

4. Gao, L. et al. Magnetic Actuation of Flexible Microelectrode Arrays for Neural Activity Recordings. *NANO LETT* **19**, 8032-8039 (2019).

5. Pas, J. et al. A bilayered PVA/PLGA-bioresorbable shuttle to improve the implantation of flexible neural probes. *J NEURAL ENG* **15**, 65001 (2018).

6. Wang, X. et al. A Parylene Neural Probe Array for Multi-Region Deep Brain Recordings. *J MICROELECTROMECH S* **29**, 499-513 (2020).

7. Srikantharajah, K. et al. Minimally-invasive insertion strategy and in vivo evaluation of multi-shank flexible intracortical probes. *Sci Rep* **11**, 18920 (2021).

8. Lu, L. et al. Soft and MRI Compatible Neural Electrodes from Carbon Nanotube Fibers. *NANO LETT* **19**, 1577-1586 (2019).

9. Vitale, F., Summerson, S.R., Aazhang, B., Kemere, C. & Pasquali, M. Neural stimulation and recording with bidirectional, soft carbon nanotube fiber microelectrodes. *ACS NANO* **9**, 4465-4474 (2015).

10. Canales, A. et al. Multifunctional fibers for simultaneous optical, electrical and chemical interrogation of neural circuits in vivo. *NAT BIOTECHNOL* **33**, 277-284 (2015).
